# Investigating Frailty, Polypharmacy, Malnutrition, Chronic Conditions, and Quality of Life in Older Adults: Large Population-Based Study

**DOI:** 10.2196/50617

**Published:** 2024-10-11

**Authors:** Yunmei Liu, Lei Huang, Fei Hu, Xiuwen Zhang

**Affiliations:** 1 School of Cultural Heritage and Information Management Shanghai University Shanghai China; 2 Department of Gastroenterology, National Clinical Research Center for Digestive Diseases, Shanghai Institute of Pancreatic Diseases The First Affiliated Hospital of Naval Medical University/Changhai Hospital, Naval Medical University Shanghai China; 3 National Key Laboratory of Immunity and Inflammation, Changhai Clinical Research Unit The First Affiliated Hospital of Naval Medical University/Changhai Hospital, Naval Medical University Shanghai China; 4 Department of General Surgery Feidong People’s Hospital, East District of the First Affiliated Hospital of Anhui Medical University Hefei China; 5 School of Clinical Medicine Anhui Medical University Hefei China

**Keywords:** statistical analyses, data mining, older adults, geriatric syndromes, frailty, polypharmacy, malnutrition, chronic conditions, quality of life, large population-based study

## Abstract

**Background:**

Aging, a significant public health issue, is associated with multiple concurrent chronic diseases and aging-related conditions (geriatric syndromes).

**Objective:**

This study aims to investigate the impact of age and chronic conditions on geriatric syndromes and the intercorrelations between multiple geriatric syndromes and quality of life (QoL) in older adults (aged ≥65 years) at the population level.

**Methods:**

A large representative sample was randomly selected from a county in China, Feidong, with 17 towns and 811,867 residents. Multiple chronic conditions, geriatric syndromes (frailty, polypharmacy, and malnutrition), and QoL were assessed and compared. Associations of demographic information and chronic conditions with geriatric conditions and QoL in older adults were assessed using multivariable-adjusted logistic regression. Intercorrelations between age, multiple geriatric syndromes, and QoL were investigated using both correlation analysis and restricted cubic splines–based multivariable-adjusted dose-response analysis.

**Results:**

Older adults comprised 43.42% (3668/8447) of the entire study population. The prevalence of frailty, premalnutrition or malnutrition, polypharmacy, and impaired QoL (median age 73, IQR 69-78 years; 1871/3668, 51% men) was 8.26% (303/3668), 15.59% (572/3668), 3.22% (118/3668), and 10.8% (396/3668), respectively. Different age and sex subgroups mostly had similar prevalence of geriatric syndromes (except that frailty occurred more often with older age). Premalnutrition or malnutrition were associated with a lower frequency of obesity and a higher frequency of constipation, polypharmacy with a higher frequency of diabetes and constipation, frailty with a higher frequency of constipation and hernia, and impaired QoL with a higher frequency of hypertension, diabetes, physical disability, and constipation. Mini Nutritional Assessment–Short Form, Groningen Frailty Indicator, and EQ-5D-5L scores, as well as the number of medications used, mostly predicted each other and QoL. Impaired QoL was associated with a higher frequency of frailty, premalnutrition or malnutrition, and polypharmacy, and frailty with a higher frequency of premalnutrition or malnutrition and polypharmacy. At a 1.5-year follow-up, impaired QoL was linked to polypharmacy and frailty at baseline, premalnutrition or malnutrition and polypharmacy were associated with frailty at baseline, and frailty was linked to both premalnutrition or malnutrition and polypharmacy at baseline. Causal mediation analyses showed that frailty mediated the link between polypharmacy and worse QoL and that polypharmacy mediated the link between frailty and worse QoL.

**Conclusions:**

In this large population-based study of older adults, multiple chronic conditions were associated with ≥1 of the investigated geriatric syndromes. Geriatric syndromes were mostly intercorrelated with, and well predictive of, each other and QoL; and causal relationships existed between geriatric syndromes and QoL, with other geriatric syndromes being mediators. The findings might be biased by residual confounding factors. It is important to perform personalized geriatric syndrome assessments stratified by chronic condition; active prevention of, or intervention for, any syndrome might help to reduce the others and improve QoL.

## Introduction

### Background

Aging is a natural process that can hardly be avoided or reversed and represents a prominent global issue. It is characterized by the functional decline of tissues and organs, increased risks of aging-associated disorders, and several biological hallmarks, including genomic instability and telomere attrition [1,2]. By 2040, the global average life expectancy is expected to increase to approximately 80 years, while the number of older adults (aged ≥65 years) is increasing in many countries, including China, potentially leading to various socioeconomic burdens and posing a significant challenge to sustainable development [3,4]. Notably, adults aged ≥85 years are the fastest-growing age group.

Huge heterogeneity exists in overall health condition and functional status (so-called physiological age) in older adults. Aging is associated with multiple concurrent chronic conditions, which may often be undertreated [4-6]. Older adults also have increased incidences of aging-related conditions (geriatric syndromes), including frailty, polypharmacy, and malnutrition, all of which may negatively impact quality of life (QoL) and limits life expectancy [7,8].

In older adults, frailty was positively linked to increased health care service use and led to higher overall costs [9-12]. Frailty might begin with a decrease in activity and the progression of exhaustion and could be mitigated by exercise [13,14]. Early identification of sarcopenia, osteoporosis, and obesity might mitigate the risk of frailty, and obesity might interact with sarcopenia in an antagonistic manner to moderate frailty rates [15-17]. Frailty was negatively linked to performance in all cognitive domains except visual perception in older adults [18]. Optimal blood pressure levels were linked to the lowest risk of frailty [19], and older adults with frailty and orthostatic hypotension had a higher mortality rate than those with frailty but without hypotension [20]. There existed an additive effect of frailty with multimorbidity on psychological stress and mortality [21-23]. Frailty was also positively linked to the severity of anemia in older adults with low dietary fiber intake [24] and impaired dentition [25]. Social frailty was associated with spousal presence, housing satisfaction, health status, and urban-rural residential differences [26]. Improving family health can help delay and reverse frailty [27].

Polypharmacy is frequent in older adults and is associated with adverse drug events; increased health care costs; cognitive and functional impairment; and increased risk of falls, hospitalizations, and mortality [28]. Polypharmacy was linked to poor medication adherence [29] and exhibited an obvious sex difference [30]. Obesity and smoking were positively associated with polypharmacy, which might cause renal and cardiovascular dysfunctions and associated mortality, especially in older adults with frailty [31-35]. The number of medications used was associated with fractures in older patients with frailty undergoing dialysis [36]. In particular, high-risk prescriptions of drugs with sedative or anticholinergic properties were linked to an increased risk of long-term care burden on society [37].

Good nutritional status was linked to high-quality sleep and an active lifestyle [38]. The prevalence of malnutrition, which was associated with anorexia or appetite loss, financial insecurity, and poor mental health, was also high in the older adult population, and special attention should be paid to older women, the oldest age groups, community-dwelling adults dependent on care, and nursing home residents [39-42]. Geriatric malnutrition could accelerate disability conditions, resulting in early functional aging [43]. It is important to regularly offer timely nutritional assessments for geriatric patients to mitigate the complications and enhance prognoses in both acute care and rehabilitation settings [44]. In the aging population, adherence to high-quality diet patterns and precision nutrition approaches were linked to a lower frequency of malnutrition [45]. In older adults, better QoL was associated with walking and strength exercises, a smaller BMI, higher education level, higher socioeconomic status, marriage, social support, and engagement in work [46-50].

### The Aims of This Study

The impact of age and chronic conditions on geriatric syndromes and the intercorrelations and causal relationships between multiple geriatric syndromes and QoL remain to be further explored and clarified [51-53]. In this large population-based study, we randomly selected a large representative sample from all residents aged ≥18 years in Feidong [51,54], a county in Hefei, the capital and largest city of Anhui province, China. We compared multiple chronic conditions, geriatric syndromes, and QoL between geriatric and nongeriatric populations and between subgroups of elderly adults and then investigated the associations of demographic information and chronic conditions with geriatric conditions and QoL in older adults. We further investigated the mutual correlations between age, multiple geriatric conditions, and QoL in older adults. Our study may offer novel useful hints for the improved care of older adults to further enhance their physical well-being and QoL.

## Methods

### Participants

**Figure 1:**
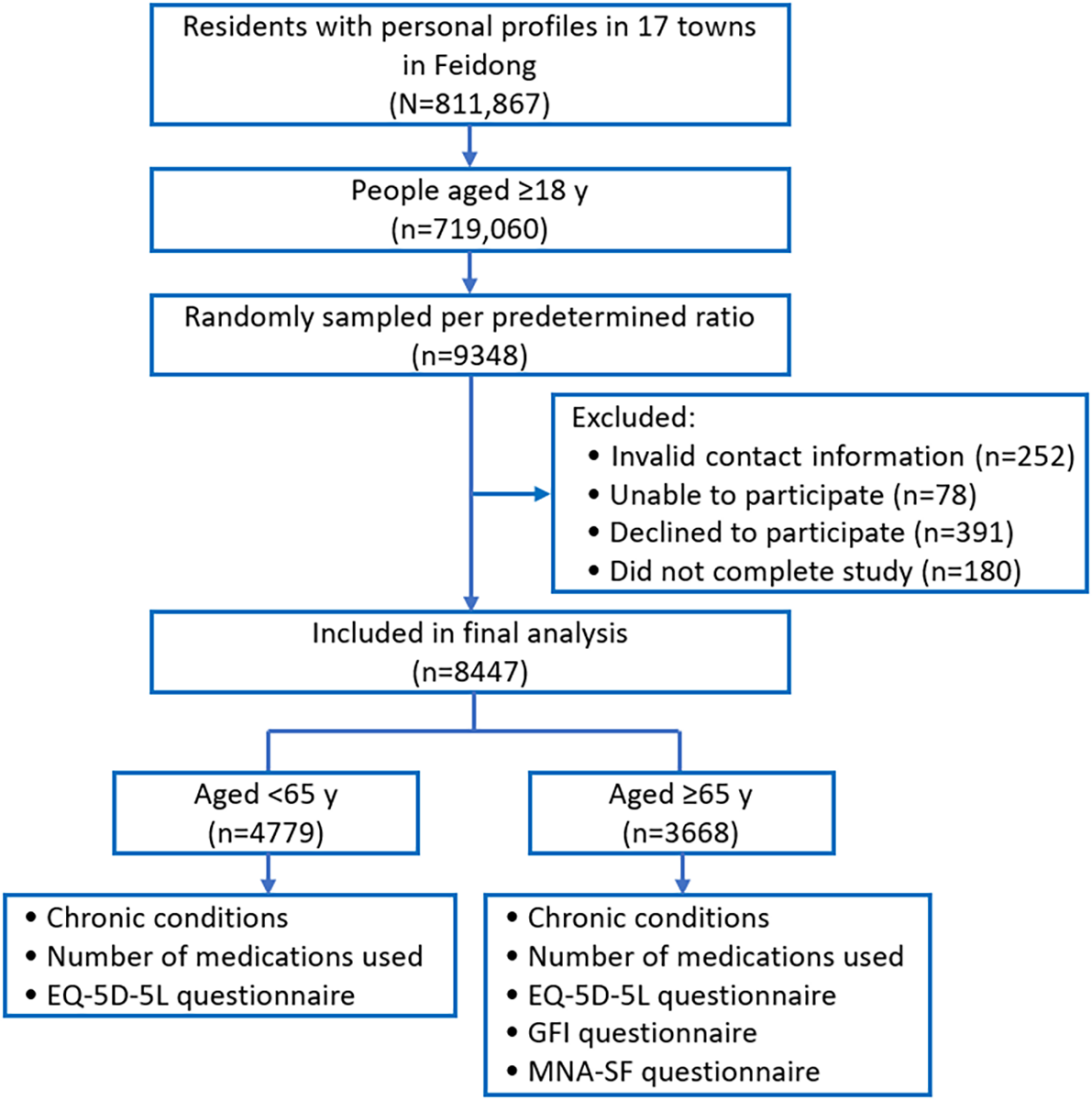
Study flow diagram. N refers to the entire population under study, and n refers to a sample population under study. GFI: Groningen Frailty Indicator; MNA-SF: Mini Nutritional Assessment–Short Form.

Participants were randomly sampled at a ratio of 1.3% (predetermined comprehensively based on estimated workload, timeline, and funding) of the whole Feidong population [51,54] who were aged ≥18 years, with stratification factors of town, sex, and age group (refer to the Sampling Ratio section in Multimedia Appendix 1). Feidong residents with complete personal profiles who were able to complete the designated questionnaires were eligible. People with invalid contact information, those who were unable or declined to participate, and those who did not complete the study were excluded after stratified random sampling (Figure 1).

All participants were queried about the number of medications used and were requested to complete the EQ-5D-5L questionnaire [55] for the assessment of QoL. Older adult participants (aged ≥65 years) were additionally assessed for frailty using the Groningen Frailty Indicator (GFI) [56] and for malnutrition using the Mini Nutritional Assessment–Short Form (MNA-SF) [57]; both instruments were specifically designed for older adults. These data were collected between October 3 and November 30, 2022.

Polypharmacy was defined as the concurrent use of ≥5 medications [58]. The GFI questionnaire was developed to evaluate frailty in older adults, and it consists of 15 items on physical (9 questions on mobility, multiple health issues, physical fatigue, vision, and hearing), cognitive (1 question), and psychosocial (5 questions on emotional isolation, depression, and anxiety) dimensions. The GFI score ranges from 0 to 15, reflecting a transition from physical well-being to severe frailty; a score of >4 indicates frailty [56]. The MNA-SF questionnaire is a validated tool used to screen older adults with malnutrition, with sensitivity, specificity, and diagnostic accuracy for predicting malnutrition being 97.9%, 100%, and 98.7%, respectively. The total MNA-SF score ranges from 0 to 14, with a score of ≤11 suggesting premalnutrition or malnutrition [57]. The EQ-5D-5L questionnaire is a validated tool used to assess QoL and includes 5 dimensions: mobility, self-care, usual activities, pain or discomfort, and anxiety or depression. The rescaled values predicted by the 8-parameter multiplicative model for China were used for computing comprehensive EQ-5D-5L scores (range –0.391 to 1.000) as recommended, with a score of 1.000 indicating instrument-defined full health [55].

Data were further linked to the personal health records database in Feidong, where information on chronic conditions with a prevalence of >1%, including hypertension, heart disease, diabetes, physical disability, constipation, hernia, and multimorbidity, was obtained for all participants. Obesity was defined as a BMI of ≥28 kg/m^2^.

### Statistics

Continuous data were presented as mean (SD) and median (IQR) and compared between 2 groups using 2-tailed *t* tests or Wilcoxon tests, as appropriate, and between 3 groups using ANOVAs or Kruskal-Wallis tests, as appropriate, followed by multiple pairwise comparisons if the overall test was significant. Categorical variables were shown as count (percentage) and compared between groups using chi-square tests or Fisher exact tests.

For the cross-sectional data without prospective follow-ups, the factors associated with premalnutrition or malnutrition, polypharmacy, frailty, and worse QoL (EQ-5D-5L score <1.000) both in the overall population and the subgroups of older adults were investigated using multivariable logistic regression. This analysis included mutual adjustment for demographic information and chronic conditions, including town, age, sex, obesity, hypertension, diabetes, physical disability, constipation, and hernia. Odds ratios (ORs) and corresponding 95% CIs were computed [59-62]. Interaction analyses of demographic information with chronic conditions were further performed in the multivariable-adjusted logistic regression models.

The intercorrelations between age as well as the EQ-5D-5L, GFI, and MNA-SF scores and the number of medications used were assessed using Pearson correlation analysis, with coefficients (*r*) computed; heat plots and chord diagrams were used to illustrate the correlations. To further reveal the interrelationships between age, geriatric syndromes, and QoL, dose-response analyses were performed using restricted cubic splines (knot number=5) [63,64]–based multivariable-adjusted logistic regression analyses to evaluate the values for age; MNA-SF, GFI, and EQ-5D-5L scores; and the number of medications used. These values were used for predicting premalnutrition or malnutrition, polypharmacy, frailty, and QoL.

We further prospectively followed up the 3668 older adults, successfully completing follow-up for 3124 (85.17%) individuals, and assessed the association of QoL at 1.5 years with geriatric syndromes, including premalnutrition or malnutrition, polypharmacy, and frailty, at baseline and of geriatric syndromes at 1.5 years with other geriatric syndromes at baseline using the log-binomial model [65] with multivariable adjustment for demographic information and chronic conditions, with relative risks (RRs) and corresponding 95% CIs calculated.

We further performed causal mediation analyses to test the hypotheses that frailty mediated the linkage between polypharmacy and worse QoL and that polypharmacy mediated the linkage between frailty and worse QoL. Data were analyzed using R statistical software (version 4.2.2; R Foundation for Statistical Computing), with 2-tailed *P*<.05 indicating statistical significance.

### Ethical Considerations

This study, as part of the Feidong Disease Surveillance Program, was approved by the ethics committee of Feidong Hospital, the eastern branch of the First Affiliated Hospital of Anhui Medical University (FDH2022073A), and written informed consent was obtained from all participants. All study data were anonymized. No compensation was offered to participants.

## Results

### Study Population

On the basis of the population census data, there were 811,867 residents with personal profiles in 17 towns in Feidong in November 2022, of whom 719,060 (88.57%; median age: 58, IQR 48-69 years; male proportion: 363,844/719,060, 50.6%) were aged ≥18 years. Per the predefined ratio, 9348 (1.3%) people were randomly selected from the eligible population of 719,060 residents; after excluding those with invalid contact information (n=252, 2.7%), those who were unable (n=78, 0.83%) or declined (n=391, 4.18%) to participate in the study, and those who did not complete the study (n=180, 1.93%), we included 8447 (90.36%) people (older adults aged ≥65 y: n=3668, 43.42%) in the final analysis (Figure 1).

The median age of the participants included in the analysis (Table 1) was 59 (IQR 46-71) years, with people aged 65 to 74 years making up the largest proportion (2247/8447, 26.6%); a little more than half of the participants were male (4278/8447, 50.65%). Hypertension (3260/8447, 38.59%), diabetes (1089/8447, 12.89%), and obesity (629/8447, 7.45%) were the most common recorded chronic conditions. Of the 8447 participants, 220 (2.6%) had polypharmacy. The mean EQ-5D-5L score was 0.986 (SD 0.079), with 8.3% (701/8447) of the participants having impaired QoL (EQ-5D-5L score <1.000).

**Table 1:** Demographic characteristics, diseases, malnutrition, polypharmacy, frailty, and quality of life of the overall population under study, people aged <65 years, and those aged ≥65 years.

Variables				Overall population (n=8447)		People aged <65 years (n=4779)		People aged ≥65 years (n=3668)		*P* values^a^	
**Demographic characteristics and diseases**											
	Age (years), mean (SD); median (IQR)		57 (20); 59 (46-71)		43 (15); 49 (31-56)		74 (7); 73 (69-78)		<.001		
	**Age group (years), n (%)**										<.001
		<45	2028 (24)		2028 (42.4)		0 (0)				
		45-54	1339 (15.9)		1339 (28)		0 (0)				
		55-64	1412 (16.7)		1412 (29.5)		0 (0)				
		65-74	2247 (26.6)		0 (0)		2247 (61.3)				
		75-84	1118 (13.2)		0 (0)		1118 (30.5)				
		≥85	303 (3.6)		0 (0)		303 (8.3)				
	Male sex, n (%)		4278 (50.7)		2407 (50.4)		1871 (51)		.56		
	BMI (kg/m^2^), mean (SD); median (IQR)		25.5 (20.1); 23.5 (21.5-26.0)		25.5 (19.5); 23.7 (21.6-26.0)		25.5 (21.1); 23.4 (21.5-25.9)		.24		
	Obesity, n (%)		629 (7.5)		399 (8.4)		230 (6.3)		*<.001* ^b^		
	Heart disease, n (%)		1908 (22.6)		882 (18.5)		1026 (28)		*<.001*		
	Hypertension, n (%)		3260 (38.6)		1147 (24)		2113 (57.6)		*<.001*		
	Diabetes, n (%)		1089 (12.9)		387 (8.1)		702 (19.1)		*<.001*		
	Physical disability, n (%)		314 (3.7)		188 (3.9)		126 (3.4)		.23		
	Constipation, n (%)		97 (1.2)		68 (1.4)		29 (0.8)		*.007*		
	Hernia, n (%)		94 (1.1)		35 (0.7)		59 (1.6)		*<.001*		
	Multimorbidity, n (%)		1620 (19.2)		641 (13.4)		979 (26.7)		*<.001*		
**Malnutrition, polypharmacy, frailty, and quality of life**											
	MNA-SF^c^ score, mean (SD); median (IQR)		—^d^		—		13 (1); 13 (12-13)		—		
	At risk of, or experiencing, malnutrition, n (%)		—		—		572 (15.6)		—		
	Polypharmacy, n (%)		220 (2.6)		102 (2.1)		118 (3.2)		*.002*		
	GFI^e^ score, mean (SD); median (IQR)		—		—		2 (2); 1 (0-2)		—		
	Frailty, n (%)		—		—		303 (8.3)		—		
	EQ-5D-5L score, mean (SD); median (IQR)		0.986 (0.079); 1.000 (1.000-1.000)		0.988 (0.075); 1.000 (1.000-1.000)		0.982 (0.085); 1.000 (1.000-1.000)		*<.001*		
	EQ-5D-5L score <1.000, n (%)		701 (8.3)		305 (6.4)		396 (10.8)		*<.001*		

^a^For comparison between people aged <65 years and those aged ≥65 years.

^b^Italicized *P* values indicate statistical significance (*P*<.05).

^c^MNA-SF: Mini Nutritional Assessment–Short Form.

^d^Not applicable.

^e^GFI: Groningen Frailty Indicator.

### Comparison of People Aged ≥65 Years and Those Aged <65 Years

In people aged ≥65 years, compared with people aged <65 years (Table 1), heart disease (1026/3668, 27.97% vs 882/4779, 18.46%; *P*<.001), hypertension (2113/3668, 57.61% vs 1147/4779, 24%; *P*<.001), diabetes (702/3668, 19.14% vs 387/4779, 8.10%; *P*<.001), hernia (59/3668, 1.61% vs 35/4779, 0.73%, *P*<.001), and multimorbidity (979/3668, 26.69% vs 641/4779, 13.41%) were more common, while obesity (230/3668, 6.27% vs 399/4779, 8.35%, *P*<.001) and constipation (29/3668, 0.79% vs 68/4779, 1.42%; *P*=.007) were slightly less frequent; the prevalence of polypharmacy (118/3668, 3.22% vs 102/4779, 2.13%; *P*=.002) was slightly higher, and the EQ-5D-5L score was negligibly lower (0.982 vs 0.988; *P*<.001), albeit with a significantly higher proportion of people having impaired QoL (396/3668, 10.80% vs 305/4779, 6.38%; *P*<.001). There were no significant differences in male proportion, BMI, or the prevalence of physical disability.

In older adults, the mean MNA-SF score was 13 (SD 1), with 15.59% (572/3668) of the people at risk of, or experiencing, malnutrition; the mean GFI score was 2 (SD 2), with 8.26% (303/3668) of the participants having frailty.

### Comparison Between Subgroups of Older Adults

In the subgroups of older adults (Table 2), the proportion of male participants was smaller in those aged ≥85 years (115/303, 38%) than in their comparators (65-74 years: 1164/2247, 51.8%; 75-84 years: 592/1118, 52.95%); the mean BMI was higher in those aged 65-74 years (26.1, SD 24.5 kg/m^2^) than in their comparators (75-84 years: 24.5, SD 14.6 kg/m^2^; ≥85 years: 24.4, SD 5.2 kg/m^2^), with the prevalence of obesity higher in those aged 65-74 years (158/2247, 7.03%) than in the other age groups (75-84 years: 56/1118, 5.01%; ≥85 years: 16/303, 5.3%); and the prevalence of hypertension was lower in those aged 65-74 years (1228/2247, 54.65%) than in their comparators (75-84 years: 702/1118, 62.79%; ≥85 years: 183/303, 60.4%). The proportions of people having frailty increased with age (65-74 years: 168/2247, 7.48%; 75-84 years: 99/1118, 8.86%; ≥85 years: 36/303, 11.9%). There were no significant differences in the prevalence of heart disease, diabetes, physical disability, constipation, hernia, or multimorbidity; in MNA-SF, GFI, or EQ-5D-5L scores; or in the proportion of people at risk of, or experiencing, malnutrition; having polypharmacy; or having impaired QoL.

**Table 2:** Demographic characteristics, malnutrition, polypharmacy, frailty, and quality of life of subgroups of people aged ≥65 years (n=3668).

Variables			People aged 65-74 years (n=2247)		People aged 75-84 years (n=1118)		People aged ≥85 years (n=303)		*P* value^a^
**Demographic characteristics and diseases**									
	Age (years), mean (SD); median (IQR)	70 (3); 70 (67-72)		79 (3); 78 (76-81)		89 (4); 88 (86-91)		*<.001* ^b^	
	Male sex, n (%)	1164 (51.8)		592 (53)		115 (38)		*.003*	
	BMI (kg/m^2^), mean (SD); median (IQR)	26.1 (24.5); 23.7 (21.8-26.0)		24.5 (14.6); 23.1 (21.1-25.4)		24.4 (5.2); 23.8 (21.9-26.0)		*<.001*	
	Obesity, n (%)	158 (7)		56 (5)		16 (5.3)		*.03*	
	Heart disease, n (%)	629 (28)		330 (29.5)		67 (22.1)		.30	
	Hypertension, n (%)	1228 (54.7)		702 (62.8)		183 (60.4)		*<.001*	
	Diabetes, n (%)	422 (18.8)		229 (20.5)		51 (16.8)		.95	
	Physical disability, n (%)	77 (3.4)		39 (3.5)		10 (3.3)		.98	
	Constipation, n (%)	18 (0.8)		11 (1)		0 (0)		.45	
	Hernia, n (%)	40 (1.8)		17 (1.5)		2 (0.7)		.17	
	Multimorbidity, n (%)	602 (26.8)		313 (28)		64 (21.1)		.27	
**Malnutrition, polypharmacy, frailty, and quality of life**									
	MNA-SF^c^ score, mean (SD); median (IQR)	13 (1); 13 (12-13)		13 (1); 13 (12-13)		13 (1); 13 (12-13)		.34	
	At risk of, or experiencing, malnutrition, n (%)	355 (15.8)		169 (15.1)		48 (15.8)		.79	
	Polypharmacy, n (%)	68 (3)		39 (3.5)		11 (3.6)		.42	
	GFI^d^ score, mean (SD); median (IQR)	2 (2); 1 (0-2)		2 (2); 1 (0-2)		2 (2); 1 (0-2)		.83	
	Frailty, n (%)	168 (7.5)		99 (8.9)		36 (11.9)		*.008*	
	EQ-5D-5L score, mean (SD); median (IQR)	0.983 (0.086); 1.000 (1.000-1.000)		0.980 (0.086); 1.000 (1.000-1.000)		0.985 (0.077); 1.000 (1.000-1.000)		.15	
	EQ-5D-5L score <1.000, n (%)	233 (10.4)		136 (12.2)		27 (8.9)		.75	

^a^For comparisons between people aged 65 to 74 years, those aged 75 to 84 years, and those aged ≥85 years.

^b^Italicized *P* values indicate statistical significance (*P*<.05).

^c^MNA-SF: Mini Nutritional Assessment–Short Form.

^d^GFI: Groningen Frailty Indicator.

### Factors Associated With Malnutrition, Polypharmacy, Frailty, and QoL in Older Adults

In people aged ≥65 years (Table 3), obesity (OR 0.17, 95% CI 0.09-0.36) was associated with a lower frequency of premalnutrition or malnutrition (MNA-SF score ≤11), while people with constipation (OR 3.32, 95% CI 1.53-7.20) had a higher frequency of premalnutrition or malnutrition. People with diabetes (OR 3.52, 95% CI 2.36-5.25) or constipation (OR 3.71, 95% CI 1.06-12.96) had a higher frequency of polypharmacy. Compared to people aged 65-74 years, those aged ≥85 years had a higher frequency of frailty (OR 1.68, 95% CI 1.11-2.55), and constipation (OR 4.88, 95% CI 1.93-12.30) and hernia (OR 2.12, 95% CI 1.05-4.29) were associated with a higher frequency of frailty, while the other analyzed chronic conditions were not. Most of the analyzed chronic conditions (hypertension: OR 1.34, 95% CI 1.06-1.71; diabetes: OR 1.31, 95% CI 1.01-1.70; physical disability: OR 2.50, 95% CI 1.56-4.00; and constipation: OR 4.11, 95% CI 1.74-9.73) were associated with lower QoL (EQ-5D-5L score <1.000). Age group was not significantly associated with premalnutrition or malnutrition, polypharmacy, or QoL in older adults. Male individuals did not significantly differ from female individuals in all outcomes of interest.

**Table 3:** Factors associated with malnutrition, polypharmacy, frailty, and quality of life of the overall group and subgroups of people aged ≥65 years, assessed using multivariable-adjusted logistic regression analyses.

Variable			Premalnutrition or malnutrition (MNA-SF^a^ score ≤11 vs >11), OR^b^ (95% CI)^c^	Polypharmacy (yes vs no), OR (95% CI)	Frailty (yes vs no and GFI^d^ score >4 vs ≤4), OR (95% CI)	Quality of life (EQ-5D-5L score <1.000 vs 1.000), OR (95% CI)
**Overall group**						
	**Age group (years)**					
		65-74	1.00 (reference)	1.00 (reference)	1.00 (reference)	1.00 (reference)
		75-84	0.95 (0.78-1.17)	1.11 (0.73-1.68)	1.23 (0.93-1.62)	1.14 (0.90-1.44)
		≥85	0.99 (0.71-1.40)	1.22 (0.62-2.42)	*1.68*^e^ (1.11-2.55)	0.85 (0.55-1.32)
	Sex (female vs male)		0.92 (0.77-1.11)	1.13 (0.77-1.66)	0.89 (0.69-1.15)	1.06 (0.85-1.32)
	Obesity (yes vs no)		*0.17* (0.09-0.36)	1.07 (0.51-2.27)	1.42 (0.91-2.22)	1.27 (0.84-1.92)
	Hypertension (yes vs no)		0.84 (0.69-1.02)	1.50 (0.95-2.36)	1.23 (0.93-1.62)	*1.34* (1.06-1.71)
	Diabetes (yes vs no)		0.85 (0.66-1.09)	*3.52* (2.36-5.25)	1.01 (0.73-1.39)	*1.31* (1.01-1.70)
	Physical disability (yes vs no)		0.77 (0.44-1.36)	0.69 (0.23-2.13)	1.55 (0.85-2.81)	*2.50* (1.56-4.00)
	Constipation (yes vs no)		*3.32* (1.53-7.20)	*3.71* (1.06-12.96)	*4.88* (1.93-12.30)	*4.11* (1.74-9.73)
	Hernia (yes vs no)		1.34 (0.70-2.57)	0.79 (0.16-3.89)	*2.12* (1.05-4.29)	1.23 (0.58-2.59)
**People aged 65-74 years**						
	Sex (female vs male)		1.01 (0.80-1.27)	1.24 (0.74-2.07)	0.75 (0.54-1.06)	1.02 (0.77-1.35)
	Obesity (yes vs no)		*0.15* (0.06-0.37)	1.23 (0.51-2.98)	1.30 (0.73-2.31)	1.32 (0.79-2.19)
	Hypertension (yes vs no)		0.85 (0.66-1.08)	*2.54* (1.35-4.78)	1.32 (0.92-1.91)	1.35 (1.00-2.82)
	Diabetes (yes vs no)		0.93 (0.68-1.27)	*2.42* (1.41-4.15)	1.27 (0.85-1.91)	*1.41* (1.02-1.97)
	Physical disability (yes vs no)		0.51 (0.23-1.13)	0.26 (0.03-2.12)	*2.21* (1.04-4.71)	*2.33* (1.24-4.39)
	Constipation (yes vs no)		*4.82* (1.82-12.77)	*5.23* (1.08-25.36)	*4.49* (1.37-14.70)	*3.97* (1.25-12.64)
	Hernia (yes vs no)		1.68 (0.78-3.60)	NE^f^	2.24 (0.98-5.13)	1.13 (0.45-2.84)
**People aged 75-84 years**						
	Sex (female vs male)		0.77 (0.55-1.08)	1.02 (0.51-2.01)	1.02 (0.65-1.59)	1.05 (0.72-1.53)
	Obesity (yes vs no)		0.32 (0.10-1.04)	0.51 (0.07-3.95)	1.76 (0.78-3.99)	1.26 (0.58-2.74)
	Hypertension (yes vs no)		0.71 (0.50-1.01)	0.89 (0.41-1.93)	1.27 (0.77-2.08)	*1.58* (1.02-2.45)
	Diabetes (yes vs no)		*0.60* (0.37-0.98)	*5.58* (2.68-11.60)	0.78 (0.43-1.41)	*1.61* (1.04-2.50)
	Physical disability (yes vs no)		1.00 (0.37-2.68)	1.08 (0.21-5.41)	0.76 (0.21-2.76)	*2.76* (1.24-6.14)
		Constipation (yes vs no)	1.79 (0.39-8.30)	4.51 (0.54-37.73)	*6.98* (1.41-34.56)	*5.53* (2.36-12.51)
		Hernia (yes vs no)	0.59 (0.13-2.82)	0.57 (0.03-10.48)	2.08 (0.45-9.60)	1.98 (0.51-7.69)
**Male individuals**						
	**Age group (years)**					
		65-74	1.00 (reference)	1.00 (reference)	1.00 (reference)	1.00 (reference)
		75-84	1.07 (0.82-1.41)	1.22 (0.67-2.22)	1.13 (0.79-1.63)	1.13 (0.82-1.56)
		≥85	0.98 (0.57-1.68)	1.52 (0.50-4.58)	1.21 (0.61-2.41)	0.61 (0.27-1.37)
	Obesity (yes vs no)		*0.11* (0.03-0.35)	1.56 (0.58-4.16)	1.59 (0.88-2.88)	1.17 (0.65-2.11)
	Hypertension (yes vs no)		*0.74* (0.57-0.97)	1.90 (0.95-3.78)	1.09 (0.76-1.58)	1.20 (0.86-1.67)
	Diabetes (yes vs no)		0.86 (0.60-1.23)	*2.90* (1.60-5.25)	1.31 (0.85-2.01)	*1.59* (1.10-2.30)
	Physical disability (yes vs no)		0.51 (0.20-1.30)	1.70 (0.48-6.02)	1.88 (0.85-4.13)	*2.22* (1.13-4.37)
	Constipation (yes vs no)		*7.87* (2.21-28.00)	*1.86* (2.95-116.86)	*4.99* (1.1.9-21.00)	*12.27* (3.46-43.57)
	Hernia (yes vs no)		1.63 (0.70-3.80)	NE	2.20 (0.88-5.49)	0.55 (0.15-1.97)
**Female individuals**						
	**Age group (years)**					
		65-74	1.00 (reference)	1.00 (reference)	1.00 (reference)	1.00 (reference)
		75-84	0.80 (0.59-1.09)	1.08 (0.61-1.92)	1.42 (0.95-2.12)	1.26 (0.90-1.75)
		≥85	1.02 (0.65-1.58)	1.13 (0.48-2.69)	*2.19* (1.30-3.69)	1.11 (0.66-1.89)
	Obesity (yes vs no)		*0.24* (0.10-0.60)	0.80 (0.24-2.67)	1.19 (0.59-2.40)	1.38 (0.77-2.49)
	Hypertension (yes vs no)		0.96 (0.73-1.28)	1.28 (0.70-2.35)	1.28 (0.87-1.90)	*1.61* (1.14-2.28)
	Diabetes (yes vs no)		0.92 (0.65-1.29)	*4.87* (2.81-8.42)	0.98 (0.63-1.51)	1.37 (0.97-1.94)
	Physical disability (yes vs no)		1.08 (0.55-2.12)	0.36 (0.05-2.68)	1.35 (0.62-2.96)	*2.69* (1.48-4.86)
	Constipation (yes vs no)		*4.22* (1.54-11.54)	3.50 (0.70-17.60)	*5.13* (1.49-17.68)	2.77 (0.84-9.15)
	Hernia (yes vs no)		1.16 (0.42-3.23)	2.30 (0.47-11.32)	2.03 (0.64-6.42)	*2.51* (1.01-6.26)

^a^MNA-SF: Mini Nutritional Assessment–Short Form.

^b^OR: odds ratio.

^c^ORs with 95% CIs for factors associated with malnutrition, polypharmacy, frailty, and quality of life were calculated using multivariable logistic regression with mutual adjustment for town, age, sex, obesity, hypertension, diabetes, physical disability, constipation, and hernia. Results for the group of people aged ≥85 years are not shown because the models did not converge for this subgroup due to a small number of cases.

^d^GFI: Groningen Frailty Indicator.

^e^OR point estimates in italics have statistical significance (*P*<.05).

^f^NE: not estimable.

The findings in the subgroups of people aged 65 to 74 years and those aged 75 to 84 years as well as in the subgroups of male individuals and female individuals were mostly similar to the overall findings in older adult participants, with a few exceptions (Table 3). In people aged 65 to 74 years, physical disability was associated with a higher frequency of frailty (OR 2.21, 95% CI 1.04-4.71), and hypertension was associated with a higher frequency of polypharmacy (OR 2.54, 95% CI 1.35-4.78). In people aged 75 to 84 years, diabetes was associated with a higher frequency of premalnutrition or malnutrition. In male individuals, hypertension was associated with a lower frequency of premalnutrition or malnutrition; and in female individuals, hernia was linked to a higher frequency of impaired QoL. Other differences in statistical significance were likely mostly due to changes in sample size.

Interaction analyses did not identify any positive interactions between demographic information and chronic conditions in the multivariable-adjusted logistic regression analyses for factors associated with geriatric syndromes and QoL (Table S1 in Multimedia Appendix 1).

Sensitivity analyses, performed by excluding hypertension and physical disability from the association analysis with frailty, did not meaningfully alter the original findings (Table S2 in Multimedia Appendix 1).

### Correlations Between Malnutrition, Polypharmacy, Frailty, and QoL in Older Adults

**Figure 2:**
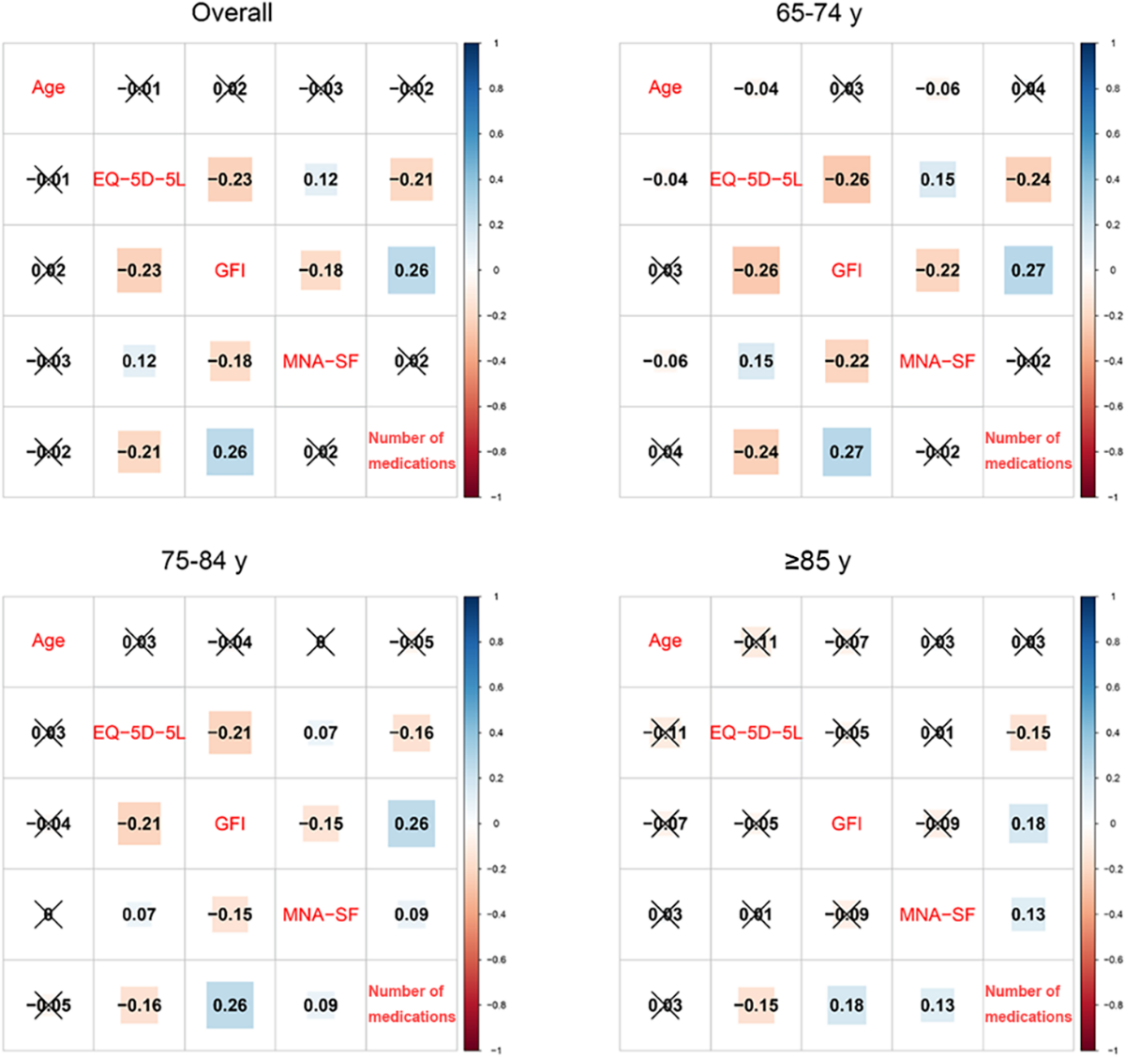
Correlation plots. Correlations are presented between age, EQ-5D-5L score, Groningen Frailty Indicator (GFI) score, Mini Nutritional Assessment–Short Form (MNA-SF) score, and the number of medications used in the overall group of older adults and in the subgroups of people aged 65 to 74 years, 75 to 84 years, and ≥85 years. The numbers in the squares represent the correlation coefficients; insignificant correlations have been crossed out.

In the overall group of older adult participants (Figure 2; Figure S1 in Multimedia Appendix 1), age was not significantly correlated with the EQ-5D-5L, GFI, or MNA-SF scores or the number of medications used. The EQ-5D-5L score was negatively associated with the GFI score (*r*=–0.23) and the number of medications used (*r*=–0.21) and positively associated with the MNA-SF score (*r*=0.12). The GFI score was correlated negatively with the MNA-SF score (*r*=–0.18) and positively with the number of medications used (*r*=0.26).

Correlation findings in the subgroups of older adults were mostly consistent with the findings in the overall group of older adults (Figure 2; Figure S1 in Multimedia Appendix 1), with a few exceptions. In people aged 65 to 74 years, age was negatively associated with the EQ-5D-5L (*r*=–0.04) and MNA-SF (*r*=–0.06) scores. In both people aged 75 to 84 years (*r*=0.09) and those aged ≥85 years (*r*=0.13), the MNA-SF score was positively associated with the number of medications used. In those aged ≥85 years, the associations of the EQ-5D-5L score with the GFI and MNA-SF scores and those between the GFI and MNA-SF scores were insignificant.

### Dose-Response Analyses

Dose-response analyses were performed using restricted cubic splines–based multivariable-adjusted logistic regression. In older adults, age predicted polypharmacy (*P*=.04) but not premalnutrition or malnutrition, frailty, or QoL (Figure S2 in Multimedia Appendix 1). With older age, the risk of polypharmacy increased until age 73 years and then decreased until age 81 years, followed by another increase afterward.

**Figure 3:**
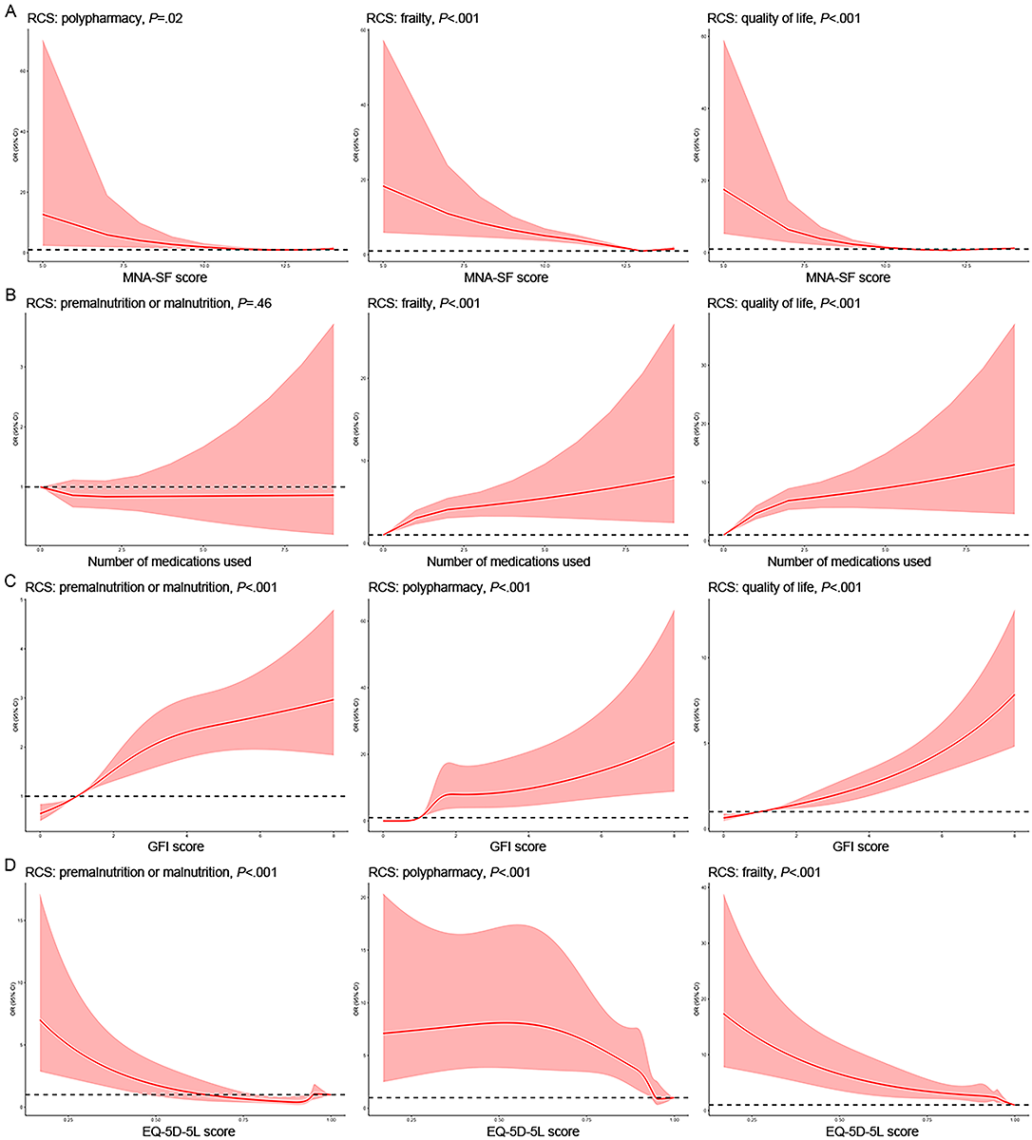
Dose-response analyses performed using restricted cubic splines (RCS)–based multivariable-adjusted logistic models. (A) Associations of the Mini Nutritional Assessment–Short Form (MNA-SF) score with risks of polypharmacy, frailty, and worse quality of life (QoL). (B) Associations of the number of medications used with risks of premalnutrition or malnutrition, frailty, and worse QoL. (C) Associations of the Groningen Frailty Indicator (GFI) score with risks of premalnutrition or malnutrition, polypharmacy, and worse QoL. (D) Associations of the EQ-5D-5L score with risks of premalnutrition or malnutrition, polypharmacy, and frailty. Red solid lines represent point estimates of odds ratios (ORs), red shadows represent 95% CIs, and black dashed lines represent an OR of 1.

With a higher MNA-SF score indicating better nutritional status, the risks of polypharmacy (*P*=.02), frailty (*P*<.001), and worse QoL (*P*<.001) all significantly decreased, especially for scores <12 (Figure 3A). The number of medications used did not significantly predict premalnutrition or malnutrition; as the number of medications used increased, the risks of frailty and poorer QoL (both *P*<.001) significantly increased, especially within the range of 0 to 2 medications (Figure 3B). A higher GFI score, indicating greater frailty, significantly predicted higher risks of premalnutrition or malnutrition, polypharmacy, and worse QoL (all *P*<.001; Figure 3C); and a higher EQ-5D-5L score, indicating better QoL, significantly predicted lower risks of premalnutrition or malnutrition, polypharmacy (for scores >0.500), and frailty (all *P*<.001; Figure 3D).

### Follow-Up Analyses

At 1.5 years of follow-up (Table 4), impaired QoL (EQ-5D-5L score <1.000) was linked to polypharmacy (RR 2.58, 95% CI 1.91-3.48) and frailty (RR 2.67, 95% CI 2.15-3.32) at baseline, but it was not significantly associated with premalnutrition or malnutrition at baseline. Premalnutrition or malnutrition at 1.5 years was associated with frailty (RR 2.15, 95% CI 1.78-2.60) at baseline, but it was not significantly linked to polypharmacy at baseline. Polypharmacy at 1.5 years was also associated with frailty (RR 2.84, 95% CI 1.83-4.37) at baseline, but it was not significantly linked to premalnutrition or malnutrition at baseline. Frailty at 1.5 years was linked to premalnutrition or malnutrition (RR 2.32, 95% CI 1.85-2.92) and polypharmacy (RR 2.59, 95% CI 1.78-3.76) at baseline.

**Table 4:** Multivariable-adjusted associations of quality of life at 1.5 years of follow-up with geriatric syndromes at baseline and of geriatric syndromes at 1.5 years of follow-up with other geriatric syndromes at baseline in older adult participants (n=3124).

Baseline	1.5-year follow-up									
	Quality of life (EQ-5D-5L score <1.000 vs 1.000)		Premalnutrition or malnutrition (MNA-SF^a^ score ≤11 vs >11)			Polypharmacy (yes vs no)			Frailty (yes vs no and GFI^b^ score >4 vs ≤4)	
	RR^c^ (95% CI)	*P* value	RR (95% CI)	*P* value	RR (95% CI)		*P* value	RR (95% CI)		*P* value

Premalnutrition or malnutrition	0.97 (0.74-1.26)	.79	—^d^	—	1.27 (0.80-2.02)		.31	2.32 (1.85-2.92)		*<.001* ^e^
Polypharmacy	2.58 (1.91-3.48)	*<.001*	1.23 (0.83-1.83)	.30	—		—	2.59 (1.78-3.76)		*<.001*
Frailty	2.67 (2.15-3.32)	*<.001*	2.15 (1.78-2.60)	*<.001*	2.84 (1.83-4.37)		*<.001*	—		—

^a^MNA-SF: Mini Nutritional Assessment–Short Form.

^b^GFI: Groningen Frailty Indicator.

^c^RR: relative risk.

^d^Not applicable.

^e^*P* values that met the threshold for statistical significance (*P*<.05) are shown in italics.

### Causal Mediation Analyses

**Figure 4:**
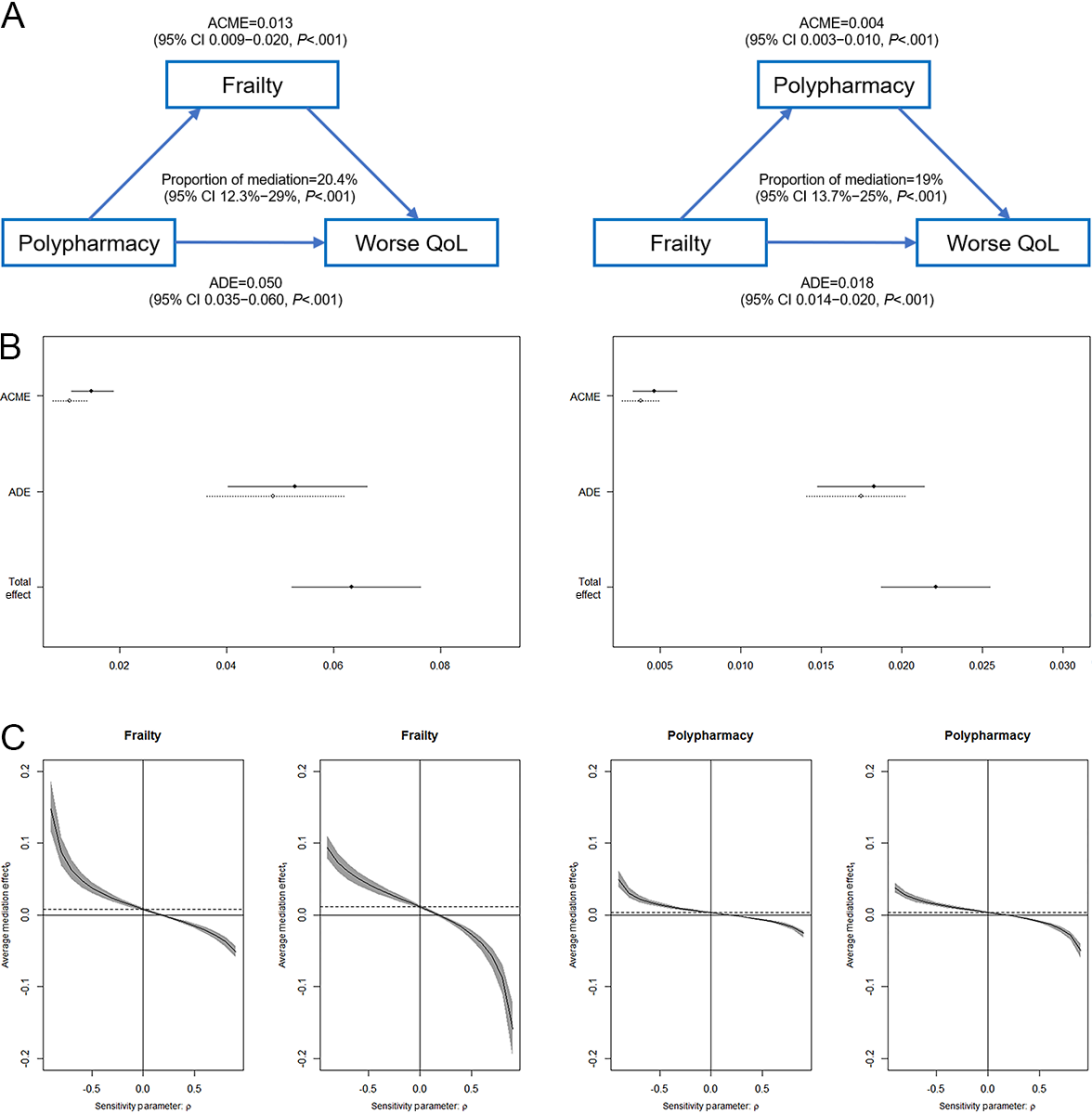
Causal mediation analyses. The hypotheses that frailty mediated the association between polypharmacy and worse quality of life (QoL) and that polypharmacy mediated the association between frailty and worse QoL were tested. (A) Schematic diagram. (B) Illustrations of average causal mediation effect (ACME), average direct effect (ADE), and total effect. (C) Sensitivity analysis.

Causal mediation analyses (Figure 4) showed that polypharmacy could directly (average direct effect=0.050, 95% CI 0.035-0.060) or indirectly (through the frailty mediator; average causal mediation effect=0.013, 95% CI 0.009-0.020) lead to worse QoL, with the proportion of mediation effect being 20.4% (95% CI 12.3%-29%), and that frailty could directly (average direct effect=0.018, 95% CI 0.014-0.020) or indirectly (through the polypharmacy mediator; average causal mediation effect=0.004, 95% CI 0.003-0.010) lead to worse QoL, with the proportion of mediation effect being 19% (95% CI 13.7%-25%).

## Discussion

### Principal Findings

Using a large randomly selected representative sample from a county in China with approximately 812,000 residents with complete personal profiles, we comprehensively reported, for the first time, the prevalence of multiple chronic conditions and geriatric syndromes; the impact of age and chronic conditions on geriatric syndromes and QoL; and the intercorrelations and causal relationships (through prospective follow-up and causal mediation analyses) between age, multiple geriatric syndromes (premalnutrition or malnutrition, polypharmacy, and frailty), and QoL in older adults at the population level.

Frailty is a reversible multidimensional syndrome characterized by reduced reserve for stressors and the accumulation of health deficits [66]. It is a prominent, typical, and heterogeneous aging-associated manifestation [27,67]. In late life, postponing or reducing frailty may help reduce health care costs [68]. Frailty is a significant risk factor for falls [69] and is strongly associated with cardiocerebrovascular disease in older Chinese people [70]. Notably, in older adults, frailty was associated with higher risks of long-term institutionalization and mortality, particularly from cardiovascular diseases [71,72]. In this study, frailty was associated with constipation and hernia but not with any of the other investigated chronic conditions in the overall older adult population [73]. Previous studies [73,74] have supported the association of constipation with frailty and poor QoL. In the subgroup of people aged 65 to 74 years, physical disability was associated with a higher frequency of frailty. Endurance and muscle strength training could help alleviate frailty in older adults with orthopedic impairment [75]. The Otago exercise program could effectively reduce frailty and improve balance and mobility in older adults [76]. A healthy lifestyle and improved family health could effectively reduce aging-related frailty [27,77]. Correlation analyses revealed that in the overall older adult population, the GFI scores were negatively associated with the EQ-5D-5L and MNA-SF scores and positively associated with the number of medications used. Multivariable-adjusted dose-response analyses indicated that higher GFI scores correlated with a higher prevalence of premalnutrition or malnutrition, polypharmacy, and impaired QoL. The ESTHER cohort study conducted in Germany [58] and the Midlife in the United States Study [78] found that frailty was associated with polypharmacy in older adults. In addition, frailty was associated with potentially inappropriate medications, and the association was restricted to certain drug classes [79]. These findings highlight the importance of the routine assessment of frailty and the implementation of corresponding public health measures to prevent, alleviate, or reverse frailty in older adults.

Polypharmacy, resulting from the need to manage multiple aging-related changes and the interplay between them, can predispose older people to increased morbidity and higher health care expenditures due to the increased risks of undesired drug-drug interactions and medication reactions [80,81]. Polypharmacy is associated with the prescription of potentially inappropriate medications and increased risks of adverse effects (which may necessitate additional drugs or hospitalization for management [82]) and falls [83]. A large German cohort study involving older adults [84] reported that polypharmacy was not independently associated with noncancer mortality. In our analysis of the overall older adult population, the number of medications used was negatively associated with the EQ-5D-5L score and positively associated with the GFI score, but it was not significantly associated with the MNA-SF score or age. Multivariable-adjusted dose-response analyses showed that a higher number of medications used predicted a higher prevalence of frailty and impaired QoL but not nutritional status. Addressing polypharmacy in older adults may reduce the impact of frailty [78]. The inverse association between polypharmacy and QoL was especially prominent during the COVID-19 pandemic [29]. People with polypharmacy had a higher frequency of diabetes and constipation. Medication use may increase the prevalence of constipation [85]. In the subgroup of people aged 65 to 74 years, hypertension was associated with a higher prevalence of polypharmacy. A Brazilian study [86] found that polypharmacy was associated with chronic noncommunicable diseases, particularly diabetes and obesity, but we did not observe a significant association between polypharmacy and obesity. These findings suggest that good management of common chronic conditions, including diabetes, hypertension, and constipation, may contribute to reducing the prevalence of polypharmacy, thus improving health-related QoL of older adults. It is vital to distinguish between appropriate and inappropriate polypharmacy [81]. This highlights the importance of appropriate deprescribing to improve QoL of older adults, which can be facilitated by multidisciplinary physician-led medication reviews and shared decision-making [87-90].

Malnutrition is common in, and a serious health risk for, older adults, and it can cause various negative consequences due to changes in body composition and function [91]. Malnutrition is associated with increased risks of various diseases and mortality in older adults [92,93]. Premalnutrition or malnutrition was associated with a lower frequency of obesity and a higher frequency of constipation. A Turkish study [94] found a positive association between constipation and malnutrition. Notably, geriatric malnutrition can accelerate disability conditions [43]. Enhancing diet, exercise, psychological support, and social support may help to address malnutrition in older adults [91]. Through correlation analyses, we found that in the overall older adult population, the MNA-SF score was positively associated with the EQ-5D-5L score and negatively associated with the GFI score. Multivariable-adjusted dose-response analyses showed that a higher MNA-SF score predicted a lower prevalence of frailty and impaired QoL. Studies from Vietnam [95] and Korea [96] have both found a strong association between nutritional status and frailty. These findings highlight the importance of screening premalnutrition or malnutrition and enhancing nutrition in older adults.

Decreased QoL is of concern, especially in older adults with chronic diseases. The presence of most of the studied chronic conditions (hypertension, diabetes, physical disability, and constipation) was associated with worse QoL. Previous studies have shown the negative association of diabetes, hypertension, and physical disability with QoL [97-99]. Constipation has been reported to be associated with a decline in word recognition, impaired QoL, and health expenses [100]. These findings highlight the importance of adequate management of chronic conditions in older adults. Engaging in adequate physical activity and improving nutrition and diet, which are associated with reduced risks of various diseases, may be easy ways to enhance QoL in older adults [101,102]. In the overall older adult population, the EQ-5D-5L score was negatively associated with the GFI score and the number of medications used and positively associated with the MNA-SF score, but it was not significantly associated with age. However, in those aged 65 to 74 years, the EQ-5D-5L score was weakly negatively associated with age. In people aged ≥85 years, the associations of the EQ-5D-5L score with the GFI and MNA-SF scores were insignificant. Multivariable-adjusted dose-response analyses revealed that a higher EQ-5D-5L score was associated with a lower prevalence of premalnutrition or malnutrition, polypharmacy, and frailty. These findings further highlighted the importance of age-stratified targeted assessments and the management of geriatric syndromes to enhance QoL in older adults.

Our findings have important implications for practice; for example, it would be important to perform routine, personalized geriatric assessments for older adults, which should be stratified by chronic condition, to precisely allocate resources and time for those truly in need. Certain chronic conditions should be carefully taken into account when screening geriatric syndromes. Our study highlights the importance of adequately addressing frailty, polypharmacy, and malnutrition in older adults. Active and targeted prevention of, and intervention for, geriatric syndromes, through good practical management of certain chronic conditions, may effectively reduce the prevalence of other geriatric syndromes and improve QoL. The prospective follow-up and causal mediation analyses suggested that active early screening and intervention for polypharmacy, frailty, and the associated chronic conditions might both directly and indirectly (through other geriatric syndrome mediators) improve QoL.

### Limitations

Our study has some limitations. First, eating and lifestyle factors were not collected. Second, the types of chronic conditions recorded in the Feidong personal health records database are somewhat limited. Some residual confounding factors not available in the data set were not accounted for and might, to some extent, impact the conclusions by introducing confounding bias. Nevertheless, we have carefully performed subgroup and stratification analyses, as well as prospective and causal mediation analyses. Heart disease and multimorbidity were registered with low sensitivity: a significant proportion of older people (especially those aged ≥85 years) with heart disease might not have had their disease registered due to not having visited a hospital or clinic; in addition, because the database might not have recorded all chronic conditions or might have recorded some with low sensitivity, the presence of multimorbidity could have been underestimated. As multimorbidity is a heterogeneous term, and different diseases might impact geriatric syndromes and QoL differently, we excluded heart disease and multimorbidity from further modeling analyses. Third, this study relied on self-reported data for medication use, which could be subject to recall bias. More objective measures, such as examinations or medical record review, could improve accuracy. However, in this population-based study, many of the participants purchased medications from retail pharmacies without visiting a hospital or clinic and without their identifying information being registered. Self-reported data are also important sources for medical research [103], and some studies [104,105] on polypharmacy are also based on self-reported data. Overall, the conclusions regarding the prevalence and determinants of polypharmacy do not differ substantially by data measurement source (self-reported surveys and prescription data) [106]. Fourth, the study population came from only 1 county in China, which limits the generalizability of the findings to other settings and populations. Multisite studies with diverse participants would improve generalizability and are warranted in the future. Fifth, while we carefully performed stratified random sampling, the sampling ratio of 1.3% was relatively small, which might influence the representativeness of the samples and introduce sampling biases. Sampling bias or error is unavoidable and is dependent on the internal heterogeneity of the whole population, sample size, and sampling method [107]. Despite the relatively small sampling ratio due to practical constraints, we conducted stratified random sampling in a single county in China, where the population would be more homogeneous, which helps to meaningfully reduce the bias. Sixth, follow-up on outcomes such as mortality and hospitalization could demonstrate the clinical effects of geriatric syndromes. Our prospective follow-up of the older adult participants captured only 2 mortalities and 7 hospitalizations, which notably could have been significantly underestimated because most of the new mortalities and hospitalizations within the follow-up period could have occurred in the older adult participants (544/3668, 14.8%) lost to follow-up. Currently, there is no readily available database to link mortality or hospitalization data.

During the data collection period, COVID-19 was still under strict control in China due to the “dynamic zeroing” policy, and there were very few analyzed participants (2/8447, 0.02%) infected with COVID-19 (of the 9348 sampled individuals, 9, 0.1% were infected and were excluded from analyses due to their inability or refusal to participate). COVID-19 infection has an important impact on frailty in older adults; for instance, frailty could contribute to the high vulnerability to severe clinical manifestations and deaths from COVID-19 infection among older adults [108] and was associated with greater disease severity and increased in-hospital mortality in older patients with COVID-19 infection [109-113].

Notably, some of the chronic conditions identified that were associated with geriatric syndromes (eg, hypertension and physical disability) are included in other frailty scales used commonly in routine health care assessments. In this study, we used the GFI, which does not include chronic conditions such as hypertension and physical disability.

### Conclusions

In this large population-based study with prospective follow-up, the prevalence of frailty, premalnutrition or malnutrition, polypharmacy, and impaired QoL in older adults was 8.26% (303/3668), 15.59% (572/3668), 3.22% (118/3668), and 10.8% (396/3668), respectively. The prevalence of these geriatric syndromes mostly did not significantly differ between different age subgroups of older adults. Multiple chronic conditions, including hypertension, diabetes, physical disability, constipation, and hernia, were associated with ≥1 of the investigated geriatric syndromes. Geriatric syndromes were mostly intercorrelated with, and well predictive of, each other and QoL, and causal relationships existed between geriatric syndromes and QoL, with other geriatric syndromes being mediators. The findings might be subject to some bias due to residual confounding factors not available in the data set. It is important to perform personalized geriatric syndrome assessments stratified by chronic condition; and active prevention of, or intervention for, any syndrome might help to reduce the others and improve QoL.

## Appendix

Multimedia Appendix 1 Supplementary material, including methods, figures, and tables.

## Abbreviations

GFI: Groningen Frailty Indicator
MNA-SF: Mini Nutritional Assessment–Short Form
OR: odds ratio
QoL: quality of life
RR: relative risk

## Data Availability

The data sets generated and analyzed during this study are not publicly available because they were used under license.
