# Supplementary material for: Investigating Frailty, Polypharmacy, Malnutrition, Chronic Conditions, and Quality of Life in Older Adults: Large Population-Based Study
Source: JMIR Public Health Surveill. 2024 Oct 11;10:e50617. doi: 10.2196/50617 (PMC11512125; doi:10.2196/50617)
Supplement: Multimedia Appendix 1 [file publichealth_v10i1e50617_app1.docx]

**Supplementary materials**

**Supplementary Methods**

**Figure S1. Chord diagrams.** Correlations between age, EQ-5D-5L score, Groningen Frailty Indicator (GFI) score, Mini-Nutritional Assessment-Short Form (MNA-SF) score, and number of drugs used in overall elderly people and the 65-74, 75-84, and 85+ years subgroups are illustrated.

**Figure S2. Dose-response analyses.** Associations of age (years) with risks of pre-malnutrition/malnutrition, polypharmacy, frailty, and worse quality of life (QoL) were analyzed using restricted cubic spline (RCS) based on multivariable-adjusted logistic models. Red solid lines represent point estimates of odds ratio (OR), red shadows 95% confidence intervals (CIs), and black dashed lines an OR of 1.

**Table S1.** Interaction analyses of demographic information with chronic conditions for associations with malnutrition, polypharmacy, frailty, and quality of life in overall and subgroups of people aged 65 years or older, assessed using multivariable-adjusted logistic regression

**Table S2.** Factors associated with frailty in overall people aged 65 years or older, assessed using multivariable-adjusted logistic regression after excluding hypertension and physical disability as adjustment factors

**Supplementary Methods**

***Sampling ratio***

The sampling ratio 1.3% was selected based on estimated workload, timeline, and funding, which supported investigation of no more than 10,000 people. There were 719,060 residents ≥18 years with Personal Profiles in Feidong; 719060×1.4%=10,067 which is greater than 10,000, and 719,060×1.3%=9348, which was closest to but smaller than 10,000. Thus the 1.3% threshold was selected. We randomly sampled people from the whole Feidong population who were ≥18 years, with stratification factors including town, gender, and age group.


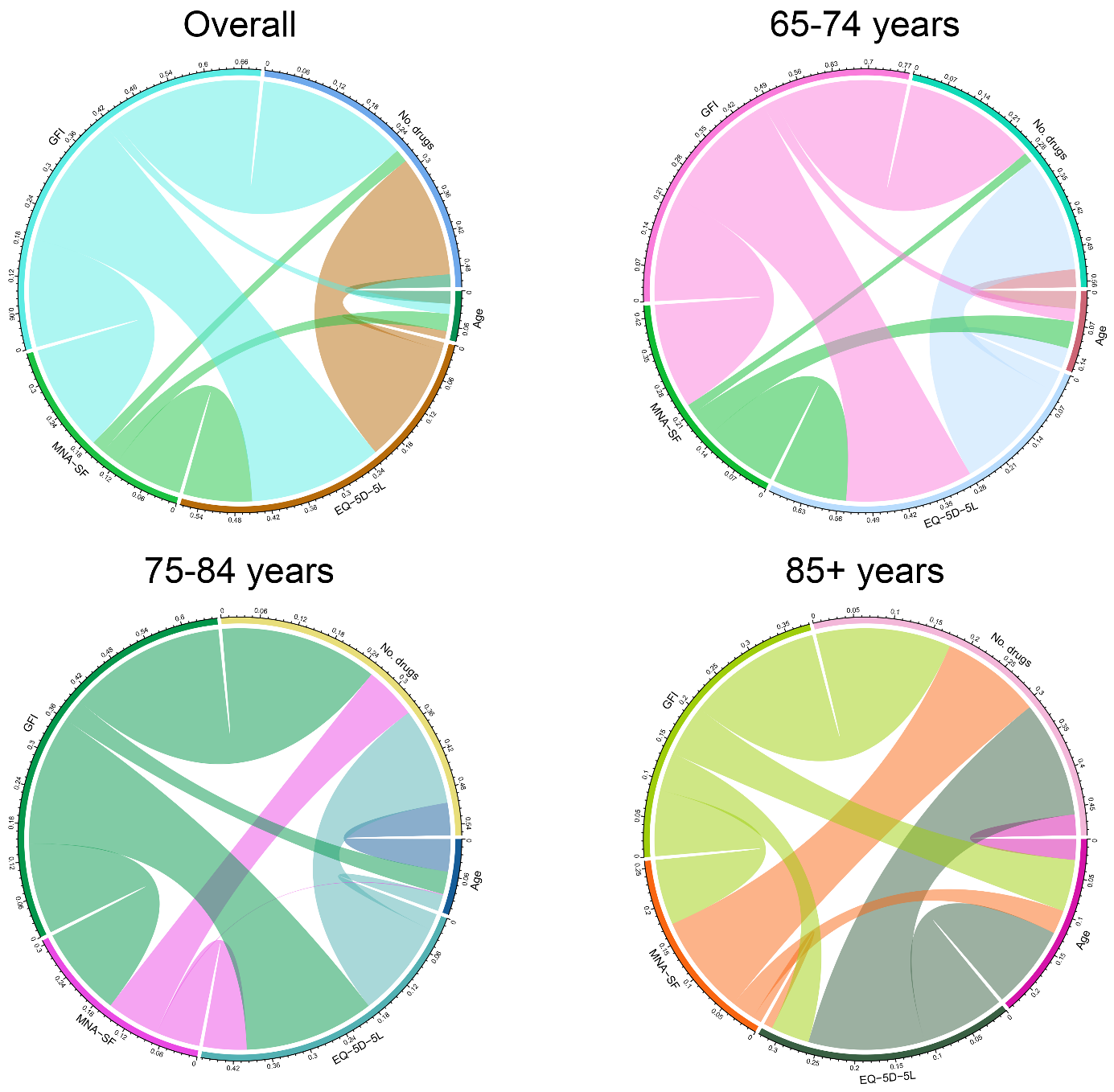


**Figure S1. Chord diagrams.** Correlations between age, EQ-5D-5L score, Groningen Frailty Indicator (GFI) score, Mini-Nutritional Assessment-Short Form (MNA-SF) score, and number of drugs used in overall elderly people and the 65-74, 75-84, and 85+ years subgroups are illustrated.


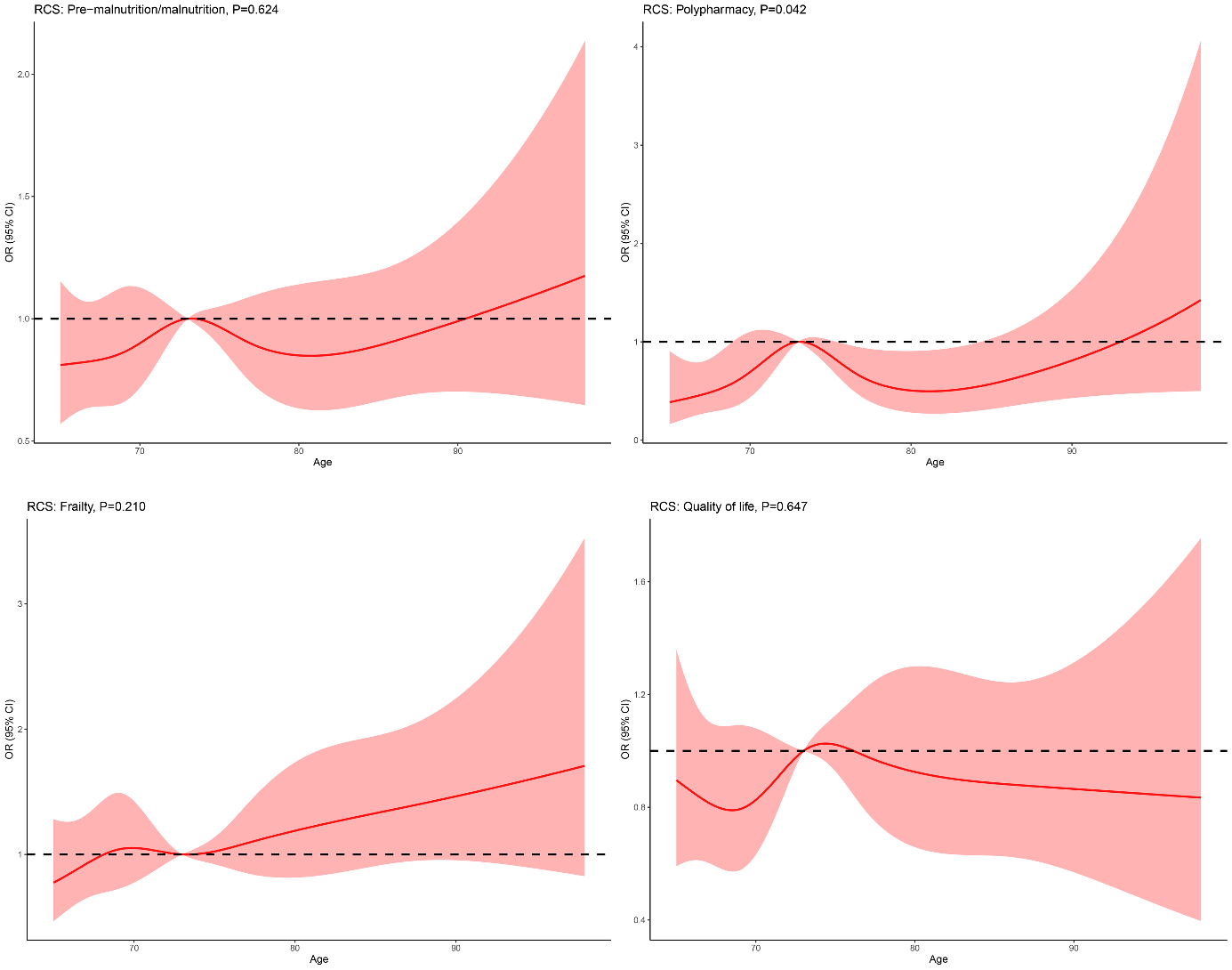


**Figure S2. Dose-response analyses.** Associations of age (years) with risks of pre-malnutrition/malnutrition, polypharmacy, frailty, and worse quality of life (QoL) were analyzed using restricted cubic spline (RCS) based on multivariable-adjusted logistic models. Red solid lines represent point estimates of odds ratio (OR), red shadows 95% confidence intervals (CIs), and black dashed lines an OR of 1.

**Table S1.** Interaction analyses of demographic information with chronic conditions for associations with malnutrition, polypharmacy, frailty, and quality of life in overall and subgroups of people aged 65 years or older, assessed using multivariable-adjusted logistic regression

| **Variable** | **(Pre-)malnutrition** (MNA-SF score ≤ vs >11) | | **Polypharmacy** (yes vs no) | | **Frailty** (yes vs no) | | **Quality of life** (EQ-5D-5L score < vs =1.000) | |
| --- | --- | --- | --- | --- | --- | --- | --- | --- |
|  | *P_interaction_* | *P_interaction_* | *P_interaction_* | *P_interaction_* | *P_interaction_* | *P_interaction_* | *P_interaction_* | *P_interaction_* |
|  | Age group | Gender | Age group | Gender | Age group | Gender | Age group | Gender |
| Obesity | 0.703 | 0.289 | 0.615 | 0.486 | 0.821 | 0.649 | 0.844 | 0.687 |
| Hypertension | 0.539 | 0.263 | 0.136 | 0.311 | 0.085 | 0.592 | 0.327 | 0.325 |
| Diabetes | 0.072 | 0.706 | 0.454 | 0.385 | 0.122 | 0.372 | 0.697 | 0.676 |
| Physical disability | 0.056 | 0.230 | 0.206 | 0.189 | 0.408 | 0.590 | 0.812 | 0.736 |
| Constipation | 0.184 | 0.631 | 0.821 | 0.373 | 0.557 | 0.924 | 0.727 | 0.108 |
| Hernia | 0.321 | 0.679 | NE | 0.984 | 0.999 | 0.868 | 0.808 | 0.064 |

NE, not estimable.

**Table S2.** Factors associated with frailty in overall people aged 65 years or older, assessed using multivariable-adjusted logistic regression after excluding hypertension and physical disability as adjustment factors

| **Variable** | **Frailty** (yes vs no) |
| --- | --- |
| ***Overall*** |  |
| Age group |  |
| 65-74 years | 1.00 (ref.) |
| 75-84 years | 1.26 (0.96-1.64) |
| 85+ years | **1.71** (1.14-2.57) |
| Gender (female vs male) | 0.89 (0.70-1.13) |
| Obesity (yes vs no) | 1.42 (0.91-2.22) |
| Diabetes (yes vs no) | 1.18 (0.87-1.58) |
| Constipation (yes vs no) | **4.74** (1.88-11.95) |
| Hernia (yes vs no) | **2.17** (1.08-4.38) |

Odds ratios (95% confidence intervals) for factors associated with frailty were calculated using multivariable logistic regression with mutual adjustment for town, age, gender, obesity, diabetes, constipation, and hernia. Results for the 85+ years group are not shown because models did not converge for that subgroup with a small case number.

NE, not estimable.
